# Evaluation of cardiovascular changes in patients with gastric wall fat halo sign

**DOI:** 10.55730/1300-0144.5420

**Published:** 2022-04-10

**Authors:** Ali KÜPELİ, Eser BULUT, Edhem ÜNVER, Gürkan DANIŞAN

**Affiliations:** 1 Department of Radiology, Faculty of Medicine, Trabzon Kanuni Training and Research Hospital, Trabzon, Turkey; 2 Department of Chest Diseases, Faculty of Medicine, Erzincan Binali Yıldırım University, Erzincan, Turkey; 3 Department of Radiology, Faculty of Medicine, Sakarya University, Sakarya, Turkey

**Keywords:** Cardiovascular disease, computed tomography, gastric wall fat halo sign, obesity

## Abstract

**Background/aim:**

This study aims to investigate the relationship between gastric wall fat halo sign and potentially associated cardiovascular disease (CVD) in thoracic computed tomography (CT).

**Materials and methods:**

Between October 2020 and January 2021, 62 patients with gastric wall fat halo sign and 62 controls were evaluated with thorax CT. Patient’s height, weight, body mass index (BMI), sex, age, comorbidities, laboratory parameters, diameters of cardiac axes, aorta and pulmonary artery; aorta and coronary artery calcium scores were recorded for the two groups.

**Results:**

No significant differences were found in sex, age, height, body weight or BMI between the two groups (p > 0.124). Patients with gastric wall fat halo sign had significantly larger diameters of the ascending aorta, the descending aorta, the main pulmonary artery, the right and left pulmonary arteries, and the short and long cardiac axes and a higher cardiothoracic ratio (CTR) than the control group (p < 0.001). Additionally, the calcium scores of the ascending, arcus, and descending aortas and the coronary arteries were significantly higher detected in patients group (p < 0.001). Patient group had significantly higher lipid profile, frequencies of diabetes mellitus (DM) and hypertension (HT) than control group (p<0.026).

**Conclusion:**

Patients with a gastric wall fat halo may show higher cardiovascular risk because of increased visceral fat tissue, vascular diameters, CTR, heart sizes, presence of DM, HT, increased lipid profile and calcium scores.

## 1. Introduction

Despite preventative and therapeutic efforts, cardiovascular diseases (CVDs), especially heart disease and stroke, are prominent causes of mortality and morbidity around the world [1]. The main risk factors and pathological mechanisms resulting in CVD can be traced back to childhood [2]. There are several controllable and uncontrollable risk factors for CVD, such as male sex, family history, old age, smoking, hypertension (HT), physical inactivity, hypercholesterolemia, diabetes mellitus (DM) and obesity [3].

The unfavorable consequences of overweight and obesity have increased worldwide and can be seen beginning in childhood [4]. Obesity is thought to be an independent risk factor for CVD, and increased body mass index (BMI) facilitates the development of comorbidities that contribute to CVD [5,6]. The generally preferred anthropometric technique to evaluate relative weight and classify obesity is BMI, which is the ratio of total body weight to height squared. Additionally, obesity is a remarkably heterogeneous condition, and *v*isceral fat tissue has been revealed to be more deeply related to CVD than subcutaneous or total fat tissue [7,8].

A fat halo sign refers to linear fat accumulation in the submucosal layer of the gastric, colonic and small bowel wall [9]. The fat halo sign can be evaluated with CT and magnetic resonance imaging (MRI). Fat halo sign is detected as fat accumulation with attenuation of <−10 Hounsfield units (HUs) on CT images and an increased fat signal in MRI [9,10]. The fat halo in the intestinal wall was first associated with chronic inflammatory bowel diseases, but it can also be commonly detected in overweight people, especially those with increased *v*isceral fat tissue [11,12]. Additionally, fat halo sign can be observed in approximately 21% of patients who underwent computed tomography (CT) examinations [11].

There are only a few studies on gastric wall fat halo sign [13–15]. To the best of our knowledge, no study has investigated the gastric wall fat halo sign as a risk factor for CVD. This paper aims to investigate the relationship between gastric wall fat halo sign and potentially associated CVD based on thorax CT.

## 2. Materials and methods

### 2.1. Participants

**Figure 1:**
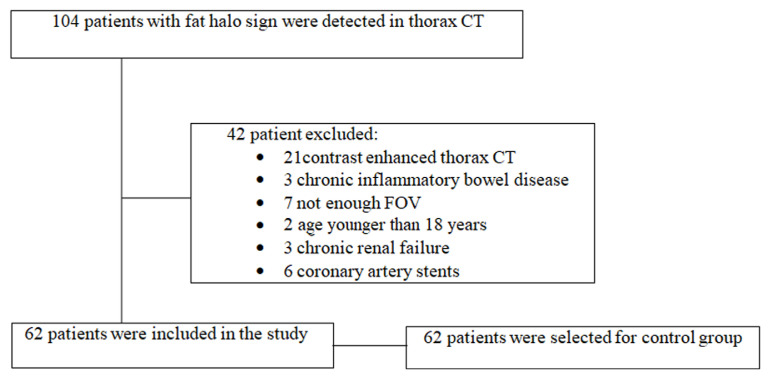
Patient flow chart.

Between October 2020 and January 2021, data from 104 patients who underwent thorax CT for clinical suspicion of COVID-19 infection and with no signs of infection in CT, negative COVID-19 PCR test, normal serum hs-CRP levels, and had gastric wall fat halo sign were prospectively recorded. Then, unenhanced thorax CT examinations in which the field of view (FOV) comprised all of the stomach were selected for study. Patients with contrast enhanced thorax CT (n = 21), chronic inflammatory bowel disease history (n = 3), not enough FOV comprising all of the stomach (n = 7), age younger than 18 years (n = 2), chronic renal failure (n = 3) and implanted coronary artery stents in which calcium score could not be measured (n = 6) were not included in the study. Therefore, 62 patients were included in the final study (Figure 1).

For the age- and sex-matched control group, 62 consecutive patients who underwent unenhanced thorax CT for clinical suspicion of COVID-19 infection and no signs of infection in CT, negative COVID-19 PCR test, normal serum hs-CRP levels and had no gastric wall fat halo sign were selected from the clinical database. Demographic and clinical data including the patients’ age, gender, height (m), body weight (kg), smoking status, comorbidities such as DM, HT and laboratory parameters such as urea, creatinine, lipid panels were noted for the patient and control groups from the medical files. Additionally, BMI (kg/m^2^) was calculated as the ratio of total body weight to height squared for both groups. This study was approved by the Institutional Ethics Committee (approval number 2021 / 17–01).

### 2.2. CT protocol

The thorax CT examinations were performed with the participants in the supine position, and the patients were scanned from the lung apices to the adrenal gland level during a breath hold at the end of inspiration with using 120 slices (Siemens Somatom Sensation, Forchheim, Germany). All patients were examined using the standard scanning protocol without intravenous contrast agent. The CT protocol was as follows: 120 kVp, tube current of 150–165 mAs, maximum 2.5 mm collimation, slice thickness of 1.5 mm and rotation time of 0.5 s. Then, the images were reconstructed into multiplanar reformations.

### 2.3. Analysis of CT images

**Figure 2:**
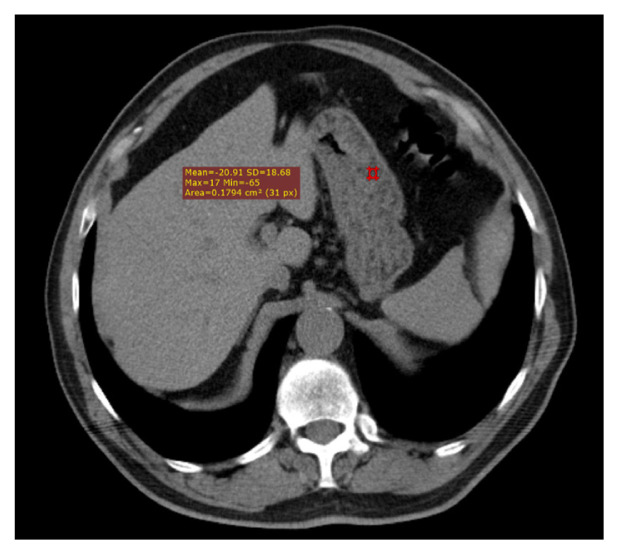
43 year-old man with back pain. Unenhanced thorax CT image represents thin linear fatty infiltration in submucosal layer of gastric wall with attenuation value <−10 HU.

**Figure 3:**
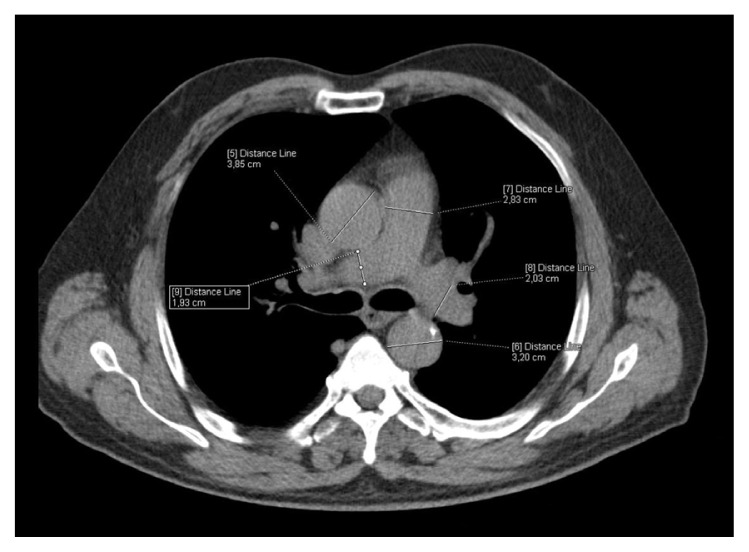
43 year-old man with cough complaint. The measurements of the ascending aorta, the descending aorta, the main pulmonary artery, and the right and left pulmonary arteries were recorded in the axial plane at the level of the pulmonary artery bifurcation.

The CT images were reevaluated by two radiologists without knowing laboratory findings and the results were obtained by consensus. The images were transferred to the Syngo workstation (Siemens Medical Solutions). Linear fat accumulation in the submucosal layer of the gastric wall with attenuation of <−10 Hounsfield units (HUs) was described as a gastric wall fat halo sign (Figure 2). The measurements of the outer wall of the ascending aorta, the descending aorta, the main pulmonary artery, and the right and left pulmonary arteries were recorded in the axial plane at the level of the pulmonary artery bifurcation (Figure 3). After detecting the axial slice level that represented the four-chamber view of the heart, the long and short cardiac axes and the maximum transverse thoracic diameters were measured at the same level ( 4). Then, the cardiothoracic ratio (CTR) was calculated as the ratio of the long diameter of the cardiac axis to the maximum transverse thoracic diameter. The calcification of the ascending, arcus, and descending aortas and coronary artery calcification were quantified with the previously defined method [16]. A region of interest (ROI) was positioned manually on the calcification of the ascending, arcus, and descending aortas and the coronary arteries. After selecting the total number of pixels over 130 HUs, the calcium score was computed with the software.

### 2.4. Statistical analysis

All of the data were analyzed using the Statistical Package for the Social Sciences (SPSS 13.0 Statistical Software, SPSS Inc., Chicago, IL, USA) and the MedCalc package of Statistical Software version 16.8 (MedCalc Software bvba, Ostend, Belgium). Descriptive statistics, including the means and ranges, were calculated for numeric variables. The Kolmogorov–Smirnov test was used to identify deviations from normal distribution and appropriate tests were selected accordingly. Additionally, the Student’s t-test was used to the compare numeric data with normal distribution. The Mann-Whitney U test was used to compare the numeric data with not normal distribution. Additionally, chi-squared tests were used to compare the categorical variables including smoking statues, HT and DM. To identify variables associated with age, the multivariate regression analysis was conducted to elucidate the predictors. Collinearity diagnostics analysis was performed to identify the potential mutilcollinearity between variables. A p value of less than 0.05 was considered to indicate a significant difference.

## 3. Results

In this study, 62 patients (45 male, 17 female) with gastric wall fat halo sign and 62 control patients (42 male, 20 female) were analyzed. The mean ages of the patient and control groups were 63.5 ± 8.1 and 61.8 ± 7.8 years, respectively. The mean height (m), body weight (kg) and BMI (kg/m^2^) of the patient and control groups were 1.62 ± 0.1 m and 1.62 ± 0.1 m; 80.8 ± 13.4 kg and 77.2 ± 12.5 kg; and 30.6 ± 4.9 and 29.4 ± 4.8 kg/m^2^, respectively. There was no statistically significant difference in sex or the mean values of age, height, body weight or BMI between the two groups (p > 0.05).

**Figure 5:**
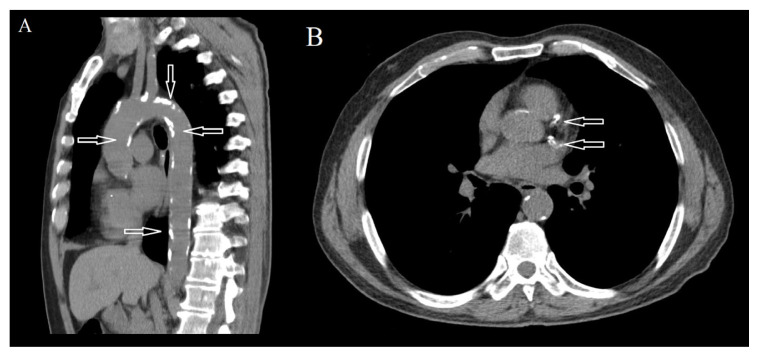
59-year-old man with gastric wall fat halo sign. Unenhanced sagittal CT image shows extensive calcific atherosclerotic plaques in aorta (A) and unenhanced axial CT image shows extensive calcific atherosclerotic plaques in coronary arteries (B).

The demographic and CT findings of patients and control groups are presented in Tables 1 and 2. Patients with gastric wall fat halo sign had significantly larger diameters of the ascending aorta, the descending aorta, the main pulmonary artery, and the right and left pulmonary arteries than the control group (p < 0.001). Although the short and long diameters of the cardiac axis and CTR were observed to be significantly higher in patients with gastric wall fat halo sign (p < 0.001), no statistically significant difference was detected in the transverse thoracic diameter between the two groups (p = 0.998). Additionally, the calcium scores of the ascending, arcus, and descending aortas (Figure 5A) and coronary arteries (Figure 5B) were significantly higher in patients with gastric wall fat halo sign than in patients without a gastric wall fat halo sign (p < 0.001).

Patients with gastric wall fat halo sign had significantly higher LDL, triglyceride, total cholesterol and lower HDL than the control group (p < 0.026). Also, no statistically significant difference was found in mean values of creatinine and BUN (p > 0.286). Although, there was no statistically significant difference in the frequencies of smoking between two groups (p = 0.443), patients with gastric wall fat halo sign had significantly higher DM and HT than without ones (p < 0.016).

The results of multivariate regression analyses performed between age and diameters of the aorta and pulmonary artery, CTR, the calcium scores of aortas and coronary arteries are shown in Table 3. There was no significant association with the primary outcome within the multivariate regression.

## 4. Discussion

In this study, the relationship between gastric wall fat halo sign and CVD was investigated in thorax CT and gastric wall fat halo sign might be detected in patients with increased aorta and pulmonary artery diameters and calcium scores.

Although there are several studies of the fat halo sign in the gastrointestinal tract, there are few studies involving the fat halo sign in the gastric wall [10–19]. To the best of our knowledge, this is the first study investigating the relationship between gastric wall fat halo sign and associated CVD based on thorax CT.

The fat halo sign was first thought to indicate the presence of inflammatory bowel disease. However, fat halo sign can be seen especially in overweight people. Additionally, gastric wall fat halo sign can be observed in patients without gastrointestinal disease and with increased visceral fat tissue [13,14]. After fat is stored in normal adipose tissues, free fatty acids are discharged into the blood [20]. The fat halo sign could be related to an increase in the blood concentration of free fatty acids and could be an indicator of excessive fat accumulation [20].

An increase in the plasma concentration of free fatty acids (FFA) is a quite often complication of visceral fat depot and overexposure of hepatic and extrahepatic tissues to FFA can cause disturbances in insulin metabolism which is resulted with insulin resistance and DM [21]. Also, obesity is related with higher LDL cholesterol, total cholesterol, triglyceride and lower HDL cholesterol plasma levels. It is reported that small dense LDL is more atherogenic in patients who have hyperlipidemia and insulin resistance associated with visceral obesity [22]. Moreover, visceral obesity leads to hypertension with complex interconnected relationship between insulin resistance, hyperlipidemia and hypertension [21]. This study showed that the frequencies of DM and HT in patients with gastric wall fat halo sign were significantly higher than control group whereas there was no significant relationship in smoking. Furthermore, patients with gastric wall fat halo sign had significantly higher LDL, triglyceride, total cholesterol and lower HDL levels than the control group. Inside this association the connection as how gastric wall fat halo sign which is consequence of visceral obesity leads to hyperlipidemia, DM and HT may be found.

The aorta and pulmonary artery diameters can be easily evaluated through chest CT. This study revealed that patients with gastric wall fat halo sign had significantly higher ascending, descending aorta and main, right and left pulmonary artery diameters than those without fat halo sign. One of the causes of these is visceral obesity. The increase in diameters associated with visceral obesity could be related to the process of adaptation to hypertension, increased blood volume and underlying structural or functional aortic abnormalities [23]. Additionally, visceral obesity can stimulate vascular aging, resulting in future cardiovascular events [8]. Visceral obesity and gastric wall fat halo sign are associated with each other, so increased aorta and pulmonary artery diameters may be related to this relationship.

Obesity is connected with most CVDs, such as coronary heart disease, hypertension and heart failure [24]. In particular, visceral adipose tissue is hormonally active tissue that contributes to vascular inflammation by producing adipokines and is a risk factor for mortality and CVD [25]. Additionally, obesity is related to some hemodynamic changes, such as increased cardiac output, diastolic filling pressure, left ventricle hypertrophy and ventricular dilatation [26]. The CTR is usually used to evaluate cardiomegaly in chest radiography, cardiomegaly indicating underlying CVD, is evidenced by hypertrophy or dilation of the heart [27]. Despite a lack of significant difference in the transverse thoracic diameter, patients with gastric wall fat halo sign had significantly higher short and long diameters of the cardiac axis and CTRs than the control group. These results may be related to cardiovascular changes caused by visceral adipose tissue.

Atherosclerosis is a major cause of death and morbidity in industrialized societies due to its association with cardiovascular events and peripheral vascular disease. Atherosclerosis has different stages, and calcification indicates advanced atherosclerotic lesions [28]. Although the cause of vascular calcification is not fully understood, inflammation, dysregulated metabolism, and osteogenesis are some processes associated with vascular calcification [29]. Thoracic aortic calcification represents a systemic atherosclerotic burden and results in an increased risk of CVDs [30]. Visceral fat tissue is an endocrine organ that can produce atherogenic agents. Additionally, this condition can induce vascular aging resulting in an increased risk of CVDs, hypertension and calcified atherosclerotic plaques [31]. It has been reported that the risk of aortic atherosclerosis increases as the amount of visceral fat tissue increases [32]. This study showed that patients with gastric wall fat halo sign had significantly higher calcium scores in the ascending, arcus, and descending aortas than patients without fat halo sign.

Coronary artery disease (CAD), caused by atherosclerosis, is a main cause of morbidity and mortality worldwide, so the early diagnosis of subclinical atherosclerosis is crucial for preventing CAD. Similar to thoracic aortic calcification, obesity is an independent risk factor for atherosclerosis and coronary artery calcification [33]. The calcium score of coronary arteries indicates the burden of atherosclerotic plaque in arteries and is a helpful, noninvasive method for the assessment of subclinical atherosclerosis in asymptomatic patients [34]. Additionally, the calcium score of coronary arteries is connected with cardiovascular events and obesity [34]. This study revealed that higher calcium scores of coronary arteries were detected in patients with gastric wall fat halo sign related to visceral obesity.

This study has a number of limitations. Atherosclerotic plaques have different components which are lipid-rich, fibrous, and calcified areas and can be often intermixed. We evaluated only calcified plaques with CT attenuation values above 130 HU. Since our CT images were obtained without intravenous contrast medium administration, the soft and intermediate plaques could not be analyzed in our study. The second limitation was that the CT images were evaluated based on a consensus, and we did not evaluate the inter- or intraobserver variability in this study. The third limitation was that participants in the patient and control groups did not undergo abdominal CT imaging, so visceral fat tissue measurement could not be performed in this study. Fourth, the number of patients was also relatively small. Fifth, data could not be correlated with echocardiographic findings.

## 5. Conclusion

The gastric wall fat halo results from excessive fat accumulation and can be observed in overweight people, especially those with increased visceral fat tissue. Additionally, patients with a gastric wall fat halo have higher cardiovascular risk because of higher frequencies of hyperlipidemia, DM, HT and increased vascular diameters, CTR, heart sizes and calcium scores. Presence of gastric wall fat halo on routine thorax or abdominal CT images may be a warning sign for cardiovascular diseases. However, extensive studies with larger populations are needed to clearly confirm the relationships of gastric wall fat halo sign with cardiovascular diseases.

## Figures and Tables

**Figure 4:**
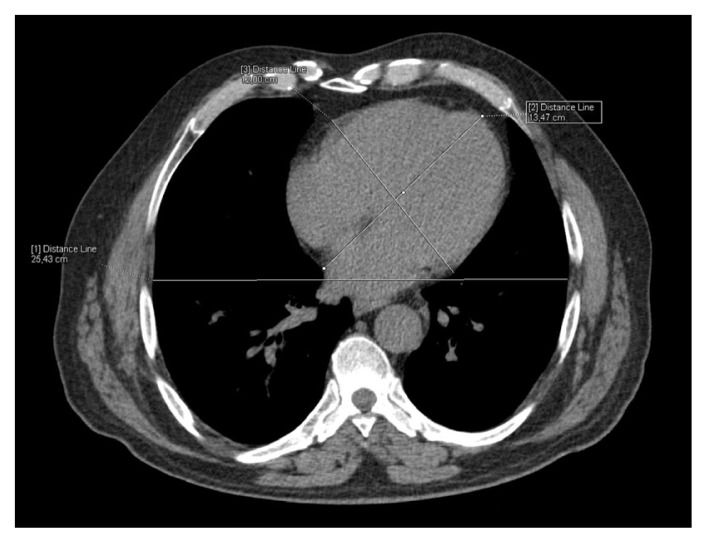
51 year-old man with headache. The measurements of the long and short cardiac axes and the maximum transverse thoracic diameter were obtained at the axial slice level that represented the maximum size of the heart.

**Table 1:** Patient characteristics.

	Gastric wall fat halo sign		p value
	Present (n = 62)	Absent (n = 62)	
Age (y)	63.5 ± 8.1	61.8 ± 7.8	0.214^a^
Sex (Male/Female)	45/17	42/20	0.556^b^
Height (cm)	162.3 ± 10.0	162.8 ± 8.9	0.779^a^
Weight (kg)	80.8 ± 13.4	77.2 ± 12.5	0.124^a^
Body mass index (kg/m^2^)	30.6 ± 4.9	29.4 ± 4.8	0.143^a^
Hypertension	32	16	0.009^b^
Diabetes mellitus	32	17	0.016^b^
Smoking	10	7	0.443^b^
Blood urea nitrogen	34.4 ± 15.6	33.2 ± 11.6	0.651^a^
Creatinine	1.0 ± 0.3	1.0 ± 0.2	0.286^a^
HDL- Cholesterol (mg/dL)	43.0 ± 10.5	48.1 ± 9.4	0.023^a^
LDL- Cholesterol (mg/dL)	119.8 ± 26.8	102.9 ± 34.8	0.026^a^
Triglyceride (mg/dL)	213.5 ± 88.3	156.1 ± 81.9	0.015^a^
Total cholesterol (mg/dL)	204.4 ± 22.8	183.1 ± 42.7	0.022^a^

Note: Values are expressed as mean ± SD,

a Student’s t-test,

b chi-squared test.

**Table 2:** CT findings of patients and control groups.

	Gastric wall fat halo sign		p value
	Present (n = 62)	Absent (n = 62)	
Aortic diameter (mm)			
Ascending aorta	38.5 ± 3.2	32.8 ± 3.9	<0.001^a^
Descending aorta	29.4 ± 2.9	24.2 ± 2.8	<0.001^a^
Pulmonary artery diameter (mm)			
Main	29.7 ± 4.4	24.4 ± 3.6	<0.001^a^
Right	22.4 ± 3.7	18.1 ± 3.1	<0.001^a^
Left	22.3 ± 2.9	17.9 ± 2.8	<0.001^a^
Long cardiac diameter (mm)	13.1 ± 1.9	11.2 ± 1.2	<0.001^a^
Short cardiac diameter (mm)	10.5 ± 1.1	9.4 ± 1.0	<0.001^a^
The maximum transverse thoracic diameter (mm)	25.2 ± 2.4	25.2 ± 2.1	0.998^a^
Cardiothoracic ratio	0.52 ± 0.08	0.44 ± 0.06	<0.001^a^
Calcium score			
Ascending aorta	0 (0–795)	0 (0–431)	<0.001^b^
Arcus aorta	237.5 (0–5064)	7 (0–1145)	<0.001^b^
Descending aorta	76.5 (0–5958)	0 (0–1145)	<0.001^b^
Total	417.0 (0–11817)	10 (0–2827)	<0.001^b^
Coronary arteries	51.5 (0–851)	0 (0–387)	<0.001^b^

Note: Values are expressed as mean ± SD or median (min-max),

a Student’s t-test,

b Mann–Whitney U test.

**Table 3:** Regression coefcients of age and variables.

	Unstandardized coefficients		Standardized coefficients	t	p value	Collinearity statistics	
	Beta	Std. error	Beta			Tolerance	VIF
Ascending aorta	0.361	0.241	0.206	1.500	0.137	0.413	2.424
Descending aorta	0.055	0.344	0.024	0.159	0.874	0.344	2.911
Main pulmonary artery	0.077	0.300	0.044	0.255	0.799	0.259	3.866
Right pulmonary artery	0.115	0.490	0.057	0.235	0.815	0.129	7.741
Left pulmonary artery	0.153	0.474	0.066	0.324	0.747	0.190	5.275
Cardiothoracic ratio	−6.914	9.992	−0.075	−0.692	0.490	0.667	1.499
Ascending aorta calcium score	0.002	0.012	0.020	0.136	0.892	0.349	2.865
Arcus aorta calcium score	−0.002	0.004	−0.166	−0.490	0.625	0.067	14.823
Descending aorta calcium score	0.002	0.002	0.170	0.992	0.323	0.274	3.648
Total	0.001	0.002	0.230	0.603	0.548	0.054	18.675
Coronary arteries calcium score	0.010	0.008	0.166	1.360	0.177	0.524	1.910
